# Transcriptional profiling of *Anopheles gambiae* mosquitoes for adult age estimation

**DOI:** 10.1111/j.1365-2583.2010.01034.x

**Published:** 2010-08-01

**Authors:** P E Cook, S P Sinkins

**Affiliations:** Department of Zoology, University of Oxford, Peter Medawar Building for Pathogen ResearchOxford, UK

**Keywords:** malaria, mosquito, vector, microarray, lifespan

## Abstract

The age distribution of female mosquitoes in the field is a critical component of vectorial capacity because of the extrinsic incubation period of mosquito-borne pathogens. However this parameter has not been well characterized in malaria vectors because of methodological difficulties; transcriptional profiling provides a potential new approach for age determination. In *Anopheles gambiae*, microarrays were used to examine global gene expression over adult life. Nine genes were selected from the 2714 gene transcripts that displayed age-related transcription patterns, and quantitative reverse transcription PCR used to select the four best performing genes. The resulting age estimation assay was able to predict female age from lab-reared samples with sufficient accuracy to provide a potentially useful tool for studies of malaria epidemiology and control.

## Introduction

One of the most sensitive parameters contributing to the vectorial capacity of malaria-transmitting *Anopheles* mosquitoes is the age distribution of wild females. After picking up the *Plasmodium* parasite there is an incubation period before transmission can occur – during which time exflagination, fertilization, zygote elongation, ookinete migration, oocyst formation and the migration of thousands of sporozoites to the salivary glands have to occur. Only then is the mosquito infectious and able to transmit malaria the next time it takes a bloodmeal. Timing of the extrinsic incubation period depends on temperature and the species of *Plasmodium*/*Anopheles*, but typically at least 9–15 days are needed; for example around 11 days for *Plasmodium falciparum* in *Anopheles gambiae* at 24 °C (Garrett-Jones, 1964; Gilles & Warrell, 2002; Smith & McKenzie, 2004), plus there is typically at least 2 days from eclosion until females take their first bloodmeal. Adult mosquitoes experience a high daily mortality rate such that only a small percentage of the total population actually survive long enough to transmit malaria (Brownstein *et al*., 2003; Cook *et al*., 2008).

Despite the critical importance of estimating *Anopheles* age in field-caught specimens to the understanding of malaria transmission dynamics and epidemiology, and the impact of control interventions, age remains one of the least understood components of vectorial capacity. Ovary dissection and microscopic examination have traditionally been used to estimate the number of gonotrophic cycles a female mosquito has gone through (Beklemishev *et al*., 1959; Detinova, 1962; Tyndale-Biscoe, 1984; Lehane, 1985; Hayes & Wall, 1999; Hugo *et al*., 2008), but this is a relatively difficult and inaccurate method and is not an absolute indicator of age because time between blood feeds can vary according to conditions and availability of human hosts for biting.

Novel control strategies to shorten mosquito lifespan are currently in development. Life-shortening *Wolbachia* bacteria from *Drosophila melanogaster* (Min & Benzer, 1997) have been successfully transferred into mosquitoes (McMeniman *et al*., 2009) with the same phenotype, thus providing an attractive control methodology. Testing of life-shortening fungal biopesticides against malaria vectors is underway (Blanford *et al*., 2005; Scholte *et al*., 2005; Bell *et al*., 2009). In addition, population age structure can be skewed toward younger individuals by current insecticidal malaria control programmes through cumulative uptake of insecticides, and it has been proposed that different insecticides should be specifically selected for delayed rather than immediate killing effects as a strategy to delay the evolution of insecticide resistance in malaria vectors (Read *et al*., 2009). Thus, the ability to estimate the age structure of natural *Anopheles* populations before and after interventions has acquired considerable importance, and there is a need to develop additional methodologies with which to do so.

A new approach developed in *Aedes aegypti* mosquitoes utilizes changes in the levels of expression of three genes in the adult mosquito (Cook *et al*., 2006, 2007). Expression changes were measured by quantitative reverse transcription PCR (qRT-PCR) on caged mosquitoes of known age and then used to build a multivariate calibration model to predict the age of field caught specimens from the same locality. This technique has been used successfully to predict age of *Ae. aegypti* from Cairns, Australia (Cook *et al*., 2006, Hugo *et al*., 2010). Here we set out to develop comparable methodologies for *An. gambiae* mosquitoes, the most important malaria vector in Africa. In order to select the most informative genes for the assay, we characterized global transcription in adult *An. gambiae* throughout adult life for both sexes. We used the *An. gambiae* genome database (Holt *et al*., 2002) to design and employ a microarray to examine global gene transcription profiles up to 30 days post-eclosion; a previous array study had examined transcription up to 15 days post-eclosion (Marinotti *et al*., 2006) but it was necessary to extend this period in order to capture expression profiles in older insects.

## Results

### Microarray results and selection of candidate genes

**Figure 1:**
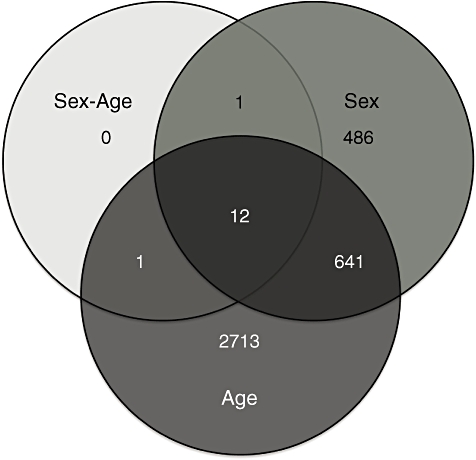
Venn diagram showing the distribution of gene transcripts that have significant differential expression with respect to age, sex and/or a sex-age interaction. A total of 3367 gene transcripts displayed significant differential expression with age; however, only 2713 transcripts did not show any significant effects with respect to sex or sex-age interaction.

Whole genome microarrays were used to identify *An. gambiae* genes that displayed age-dependent transcription levels in both male and female adult mosquitoes. A custom microarray was designed with a single probe per *An. gambiae* gene transcript (13254 probes). One-colour microarray hybridizations were run with total RNA extracted from pools of male and female mosquitoes at 0, 10, 20 and 30 days post-eclosion. Fluorescence data were quantified from the microarray scan files using Agilent Technologies' Feature Extraction software. Data processing and analysis were carried out with GeneSpring GX. Two-way analysis of variance (*P*≤ 0.01; Benjamini–Hochberg multiple test correction) identified 2713 gene transcripts that displayed significant differential expression across adult mosquito age and no significant difference resulting from sex or age–sex interaction (Fig. 1). From these, candidate genes were selected for showing a strong correlation between transcript levels and adult age/a greater than fourfold change in transcription across multiple age classes. Five transcripts, AGAP004115-RA, AGAP006187-RA, AGAP006985-RA, AGAP010398-RA and AGAP012396-RA (Fig. S1A–E, respectively), showed a strong positive correlation between transcript levels and both male and female mosquito age. Two transcripts, AGAP006829-RA and AGAP008447-RA (Fig. S1F, H, respectively), displayed a negative correlation between transcript levels and age. The final two down-regulated gene transcripts, AGAP007963-RA and AGAP009871-RA (Fig. S1G, H, respectively), were selected because they are putative orthologues of the two most important genes used in the *Ae. aegypti* transcriptional age-grading protocol (Cook *et al*., 2006). AGAP007963-RA was selected from the 2714 gene transcripts that displayed age-related transcription (ie no significant effect of sex or age-sex interaction). AGAP009871-RA had significant age- and sex-related transcription. The reference gene, 40S ribosomal protein S7 (RS7_ANOGA; AGAP10592-RA), was not differentially regulated with adult male or female mosquito age (Fig. S1J).

The transcriptional profiles of the nine candidate genes were investigated in individual adult mosquitoes using qRT-PCR. Primers were designed to amplify a 100–150 base pair region of each gene transcript (Table 1). Transcript abundance of each candidate gene was determined relative to RS7_ANOGA as a logcontrast of cycle threshold (Ct) values. A low Ct value indicates high transcript abundance and a high Ct value corresponds to a low transcript copy number. All nine candidate genes showed age-related transcriptional profiles and results were generally consistent with that observed in the microarray experiment. All up-regulated candidate genes, AGAP004115, AGAP006187, AGAP006985, AGAP010398-RA and AGAP012396, showed negative, linear associations between logcontrast Ct values and age, in both male and female mosquitoes (Fig. S2A–E, respectively). Three of the four down-regulated candidate genes, AGAP006829, AGAP007963 and AGAP009871 (Fig. S2F, G and I, respectively), displayed positive associations with logcontrast Ct values and age, in females and males. The remaining candidate gene, AGAP008447 (Fig. S2H), had a positive association across female age and an unexpected negative association with male age. Transcript AGAP008447 was subsequently excluded from the analysis because of the possible confounding effects of insecticide resistance status (see Discussion).

**Table 1:** Candidate age-grading genes selected from the microarray analysis

Transcript identifier	Gene name	Putative function
AGAP004115-RA		Cystinosin, putative
**AGAP006187-RA**	G12_ANOGA	Protein G12 precursor (ANG12)
AGAP006985-RA		Conserved hypothetical protein
**AGAP010398-RA**		Dimethylaniline monooxygenase, putative
**AGAP012936-RA**		Cystinosin, putative
AGAP006829-RA	CPR59	Cuticular protein 59
AGAP007963-RA*		Calcium-binding protein, putative
AGAP008447-RA†	CPLCG4	Adult cuticle protein
AGAP009871-RA*	CPR75	Cuticular protein 75 (RR-1 family)
AGAP010592-RA	RS7_ANOGA	40S ribosomal protein S7

Transcript identifiers and gene function originate from Vectorbase annotations. When gene function has not been annotated putative gene function has been inferred from *Drosophila melanogaster* and/or *Aedes aegypti* orthologues. The four best performing gene transcripts used for the age estimation analysis shown in Fig. 3 are in bold.

* Orthologues of genes selected for use in the *Aedes aegypti* age estimation assay (Cook *et al*., 2006).

† Gene excluded from the analysis because of possible confounding effects of insecticide resistance status (see Discussion).

**Figure 2:**
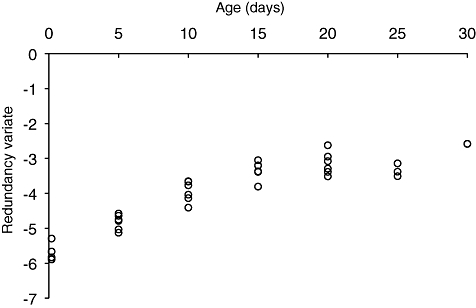
Female *Anopheles gambiae* calibration model generated from the expression profiles of the four best candidate genes. Logcontrast normalized cycle threshold values for AGAP006187, AGAP006985, AGAP007963 and AGAP010398 were entered into a canonical correlation analysis. This analysis generates a redundancy variate, a linear combination of the four gene expression measures, which maximizes correlation with mosquito age. The redundancy variate score for each individual is plotted and the linear regression of these data on age provides the calibration model.

Redundancy analysis was used to reduce multiple gene expression measures into a redundancy variate, a linear combination of gene expression measures, which maximized correlation with mosquito age. This analysis was initially carried out using all genes and from there an optimal set of genes was selected based on the canonical coefficient loadings from the redundancy analysis; more informative genes have a larger absolute loading (either positive or negative depending on the relationship between a particular gene expression profile and age). Based on these criteria, four transcripts were selected for the age estimation assay: AGAP006187, AGAP007963, AGAP010398 and AGAP012936. These genes performed very well in females generating a calibration model that was highly correlated with age (Fig. 2; *R*^2^= 0.8206, *n*= 34, *P* < 0.0001). However in males, these genes generated a redundancy variate that was not as strongly correlated with age (Fig. S3; *R*^2^= 0.6099, *n*= 35, *P* < 0.0001); the reasons for this difference are unclear.

**Figure 3:**
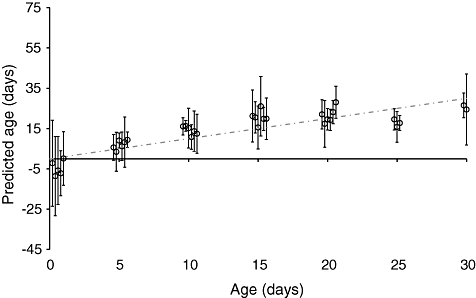
Age predictions for individual female mosquitoes using four gene expression measures. Age predictions were made using a leave-one-out cross validation approach, whereby each data point was sequentially removed from the training data before the age of the excluded individual was predicted. Age was predicted by inverse regression of the female calibration model. Error bars indicate a 95% confidence interval derived by a nonparametric bootstrap procedure.

Age predictions were derived for females using inverse regression of the calibration model constructed from the four most informative genes (Fig. 3). A leave-one-out cross validation methodology was used to generate a large number of age predictions from the available data. The precision of each age prediction was estimated using a nonparametric bootstrapping procedure that derived 95% confidence intervals (CIs). The median age within these 95% CIs was considered to be the predicted age. Predicted ages had a mean 95% CI of ± 4.2 days across all ages. The mean residual value (the absolute difference between predicted age and actual age) for females is 4.3 days. Negative ages are predicted but this was to be expected when predicted ages are close to zero simply as a result of error within the calibration model.

For males, predicted ages had a mean 95% CI of ±9.1 days across all ages (Fig. S4), and a mean residual value of 10.1 days. This higher variability was expected given the poorer fit observed in the calibration model. Four samples were notable outliers in the age predictions; however, this could not be attributed to a single disparate gene expression measure across samples – that is, it was a different gene for each specimen that caused the inaccuracy in age prediction. The distribution of the residual values when plotted against predicted age suggests that there was no bias associated with the age predictions for either sex (Fig. S5).

## Discussion

The primary purpose of these experiments was to develop an assay capable of detecting differences in female population age structure in order to be able to compare populations with and without control interventions, and the effects of age structure on malaria epidemiology. We consider the data presented to provide the baseline for an assay that would be a very useful addition to the field as the confidence intervals in individual age predictions are acceptable for making population-level comparisons. The assay was less successful for males, but the requirement for age prediction methodologies is in any case much smaller in males, which do not blood feed or transmit malaria. In addition, male age-grading methodologies are now available that use the number of spermatocysts in *An. gambiae s.s.* male testes and the relative size of their sperm reservoirs (Huho *et al*., 2006).

Three of the genes examined encoded cuticular proteins. Cuticular protein genes of low complexity (CPLC) constitute a family of at least 210 genes, including 27 in a cluster encoding family CPLCG (Cornman *et al*., 2008; Cornman & Willis, 2009). Several adult cuticular protein genes have recently been reported to show increased levels of expression associated with pyrethroid insecticide resistance (Vontas *et al*., 2007; Djouaka *et al*., 2008; Awolola *et al*., 2009), including *CPLCG4* (previously named *CPLC*#; AGAP008447). This appears to constitute a mechanism of resistance based on cuticular thickening to reduce insecticide penetration, but understanding of this mechanism is in its early stages. Because of the potential for variation in the expression of cuticular proteins associated with insecticide resistance status of populations under investigation, and because this factor is not yet well understood, the adult cuticular protein gene was excluded from the current assay. In addition variation in cuticular hydrocarbons amongst and within species in the *An. gambiae* complex (Carlson & Service, 1979, 1980; Anyanwu *et al*., 2000, 2001) could act as a further confounding variable. This variation is possibly associated with selection for mate choice, as has been shown in *Drosophila* for example (Foley *et al*., 2007).

The analysis of cuticular proteins for age estimation has been assayed in *An. gambiae* and other anophelines with varying degrees of success (Randimby *et al*., 2002; Caputo *et al*., 2005; Hugo *et al*., 2006; Mayagaya *et al*., 2009). A recently reported assay using near -infrared spectroscopy (Mayagaya *et al*., 2009) showed promise as, in the authors' words, a ‘quick and dirty’ assay with minimal sample processing costs once the initial outlay on a spectrophotometer has been made, allowing large numbers of samples to be assayed. This assay does not appear sensitive enough to estimate age differences beyond about 15 days, which is the most epidemiologically significant class. However, it is possible that a combination of assays could be used to improve throughput and reduce costs, with near-infrared spectroscopy used to assign females to ‘younger’ and ‘older’ classes, and then qRT-PCR based on the methodologies described here could be employed on the older class (which would be a minority subset). In this case, however, it would be important to examine and control for any insecticide resistance effects on cuticle composition, for the reasons outlined above.

Future experiments will examine the robustness and utility of the assay on natural populations from different geographical locations, molecular forms of *An. gambiae s.s.*, and sibling species within the *An. gambiae* complex such as *Anopheles arabiensis*. The effects on the accuracy of the age predictions of temperature variation and larval rearing conditions will also be examined; it is possible that other genes in Table 1 than those selected may prove to be more robust under different conditions. However we consider that the assay presented here provides a solid foundation for future refinement, and represents a new tool that can assist a variety of studies related to malaria epidemiology and control.

## Experimental procedures

### Mosquito rearing and collection

*An. gambiae* G3 colonies were maintained under standard insectary conditions (∼28 °C; ∼70% relative humidity; 12:2 h light : dark). Adults were given 10% sucrose solution *ad libitum*; however no bloodmeals were provided. Adults emerging within a 24 h period were considered a single age class and each age class allocated to separate cages. Adult male and female mosquitoes were collected at 0 (≤24 h), 5, 10, 15, 20, 25 and 30 days post-eclosion. Collected individuals were knocked down with ∼2 min exposure to −20 °C before the abdomen was dissected and discarded. The head and thorax were transferred directly into Trizol reagent (Invitrogen, Carlsbad, CA, USA) and stored at −80 °C.

### Microarray design

Custom gene expression arrays (8 × 15 k format) were designed using Agilent Technologies' eArray (https://earray.chem.agilent.com/earray). The microarray contained 13 254 custom probes, a single 60-mer probe per *An. gambiae* transcript (Vectorbase genebuild AgamP3.4 transcripts; http://www.vectorbase.org) and a standard set of 536 Agilent control features.

### Total RNA isolation and labelling protocol

Global transcription was quantified at 0, 10, 20 and 30 days of adult age in both male and female *An. gambiae*. Total RNA was extracted from a pool of five individuals (heads and thoraces only) in 500 µl Trizol reagent. The manufacturer's standard RNA extraction protocol was used, with the exception that RNA was precipitated by a ∼16 h incubation at −20 °C. Array experiments were performed according to Agilent Technologies' One-colour Microarray-based Gene Expression Analysis protocol (v. 5.7, Agilent Technologies, Palo Alto, CA, USA). All consumables and equipment were from Agilent Technologies unless otherwise stated. Briefly, 1 µg total RNA and 5 µl of 1:5000 dilution Agilent One-colour Spike-in mix were amplified and labelled using the Quick Amp Kit protocols (Agilent Technologies). Labelled cRNA was then purified and quantified before hybridization to the arrays using Agilent Technologies' Gene Expression Hybridization Kit, SureHyb chambers, Microarray Hybridization oven and standard protocols. Hybridized arrays were washed with Gene Expression Wash Buffers (without the addition of Triton X102) and Stabilization and Drying Solution to protect against ozone degradation.

### Microarray scanning, feature extraction and data analysis

Microarrays were scanned and feature extraction undertaken by technicians at Oxford Gene Technology (Oxford, UK). Scans were at 5 µm resolution using the extended dynamic range feature of the Agilent Technologies' DNA microarray scanner (G2505B). Feature Extraction software (v. 9.5.3.1; Agilent Technologies) was used to quantify and process fluorescence data from the array image files. This process was carried out according to Agilent's One-colour Gene Expression protocol (GE1-v5-95_Feb07). Data files were deposited in the National Center for Biotechnology Information Gene Expression Omnibus (GEO) according to Minimum Information About a Microarray Experiment (MIAME) requirements (accession GSE18194).

Array analysis was undertaken with Genespring GX software (v. 10.0.2; Agilent Technologies). The array technology file was created directly from the eArray website (see Microarray design section) using the corresponding microarray design files. Signal intensity data were processed as follows; threshold to 1.0, log_2_ transformation, 75^th^ percentile shift normalization and baseline transformation to the median of all samples. Array data were filtered by raw signal values (excluding data ≤20^th^ percentile) and flags generated during feature extraction. Flags indicated whether a feature signal was uniform, saturated and significant above background. Differentially expressed transcripts were identified by a two-way analysis of variance at *P*≤ 0.01 (asymptotic *P*-value computation) with Benjamini–Hochberg multiple testing correction. Gene transcripts that were differentially expressed only with respect to adult mosquito age were selected. These transcripts were then filtered for transcriptional profiles that were either positively (*r* > 0.8) or negatively (*r* < −0.4) correlated with mosquito age. Attention was also given to transcripts with fourfold or greater changes in transcription between the various age classes.

### Quantitative RT-PCR age-grading assay

Candidate gene and reference gene expression were measured in adult male and female mosquitoes at 0, 5, 10, 15, 20, 25 and 30 days post-eclosion using qRT-PCR.

Total RNA was extracted from the head and thorax of individual mosquitoes using 250 µl Trizol reagent. Extractions were carried out according to the manufacturer's protocol with the exception of RNA precipitation, which was a ∼16 h incubation at −20 °C. RNA was re-suspended in 20 µl RNase-free water. Total RNA was quantified using a Nanodrop ND-1000 spectrophotometer (Thermo Fisher Scientific, Waltham, MA, USA). 500 ng total RNA was treated with 1 unit of DNase I (amplification grade, Invitrogen) mixed with supplied 10× DNase buffer solution. The reaction was incubated at room temperature for 15 min before being terminated with 25 mM ethylenediaminetetraacetic acid and incubated at 65 °C for 10 min.

Reverse transcription was performed using the Superscript VILO cDNA synthesis kit (Invitrogen) according to the manufacturer's protocol. cDNA samples were diluted fivefold prior to qRT-PCR amplification to eliminate PCR inhibitors. Quantitative RT-PCRs were performed in triplicate on a Lightcycler 480 (Roche, Indianapolis, IN, USA) using Express SYBR® Greener qPCR supermix Universal (Invitrogen), 1 µl diluted cDNA template and 400 nM of each primer. Thermal cycling conditions were 95 °C for 5 min; then 45 cycles of 95 °C for 10 s, 58 °C for 10 s, 72 °C for 15 s and a single fluorescence acquisition; and then a melt curve of 95 °C for 5 s, 65 °C for 1 min, then ramp to 97 °C with continuous fluorescence acquisition (5 acquisitions/°C). Ct values were determined using the second-derivative maximum method (Roche).

### Multivariate calibration and age predictions

The statistical methods employed to predict mosquito age followed the multivariate calibration approach as previously outlined (Cook *et al*., 2006, 2007). Briefly, mean Ct values were calculated and normalized to the reference gene (RS7_ANOGA) by logcontrast ratios. Redundancy analysis, implemented through a canonical correlation analysis in SAS (SAS Institute Inc., Cary, NC, USA), was used to calculate a linear combination of gene expression measures (a redundancy variate) that maximized the correlation with mosquito age. The linear regression of the first redundancy variate on age was used to construct a calibration model. Age predictions were estimated from this calibration model by inverse regression techniques. The error associated with age predictions was estimated using a nonparametric bootstrapping procedure. In this study, calibration models were evaluated by generating age predictions using a leave-one-out cross validation approach.

## Supporting Information

**Figure S1.** Age-dependent transcription levels in both male and female adult mosquitoes. Transcription profiles of the nine candidate genes (A–I) and reference gene (J) were measured using one-colour microarrays and displayed as normalized signal intensity (log_2_) across adult female and male mosquito lifespan.

**Figure S2.** Logcontrast gene expression measures across adult male and female mosquito lifespan. Transcript abundance of each candidate gene (A–I) was determined relative to RS7_ANOGA as a logcontrast of cycle threshold (Ct) values.

**Figure S3.** Male *Anopheles gambiae* calibration model generated from the expression profiles of the four best candidate genes. Logcontrast normalized cycle threshold (Ct) values for AGAP006187, AGAP006985, AGAP007963 and AGAP010398 were entered into a canonical correlation analysis. This analysis generates a redundancy variate, a linear combination of the four gene expression measures, which maximizes correlation with mosquito age. The redundancy variate score for each individual is plotted and the linear regression of these data on age provides the calibration model.

**Figure S4.** Age predictions for individual male mosquitoes using four gene expression measures. Age predictions were made using a leave-one-out cross validation approach, whereby each data point was sequentially removed from the training data before the age of the excluded individual was predicted. Age was predicted by inverse regression of the female calibration model. Error bars indicate a 95% confidence interval derived by a nonparametric bootstrap procedure.

**Figure S5.** Residuals, the absolute differences between predicted age and actual age, for individual female (A) and male (B) mosquitoes plotted against predicted age. The distribution of the residuals suggests that there was no bias associated with age predictions.

Please note: Neither the Editors nor Wiley-Blackwell are responsible for the content or functionality of any supporting materials supplied by the authors. Any queries (other than missing material) should be directed to the corresponding author for the article.
