# Supplementary figures and images for: Transcriptional profiling of Anopheles gambiae mosquitoes for adult age estimation
Source: Insect Mol Biol. 2010 Aug 1;19(6):745–51. doi: 10.1111/j.1365-2583.2010.01034.x (PMC2998705; doi:10.1111/j.1365-2583.2010.01034.x)

Normalized signal intensity ( $\log_2$ )

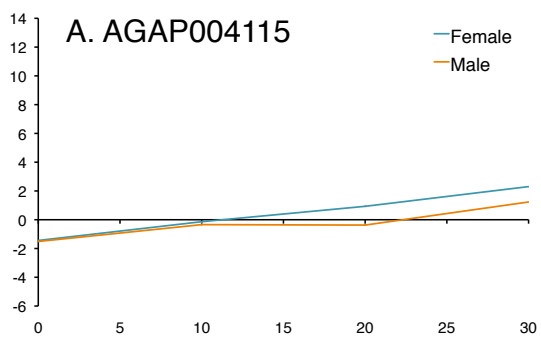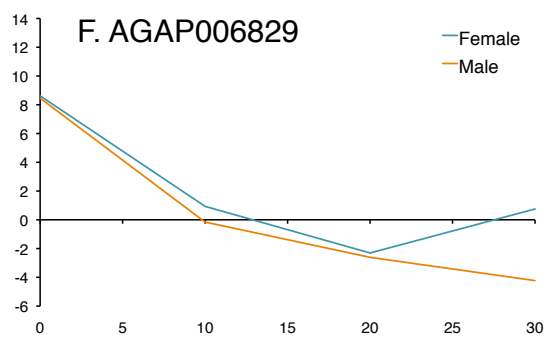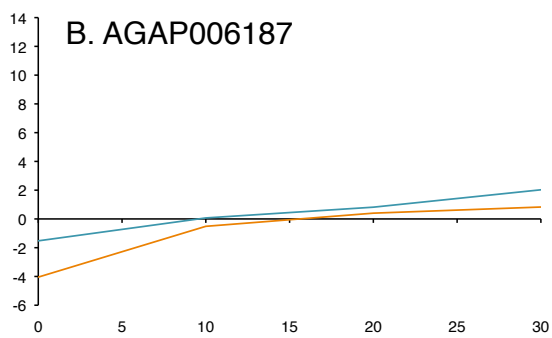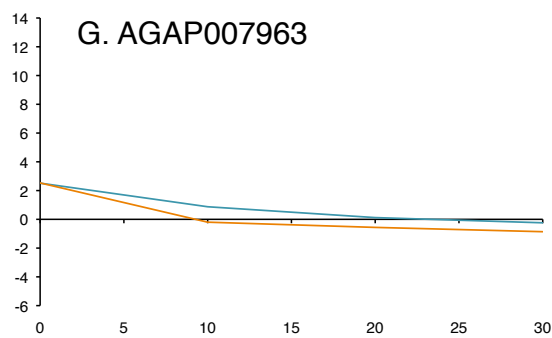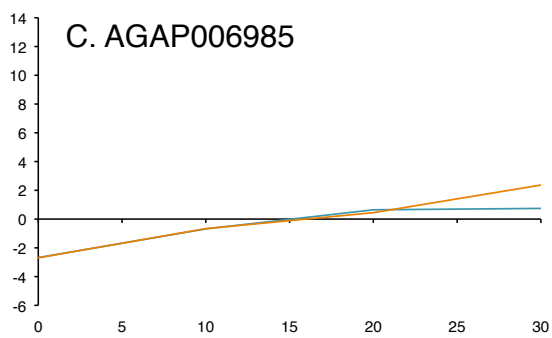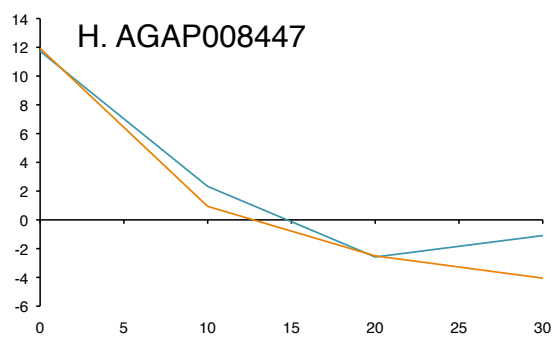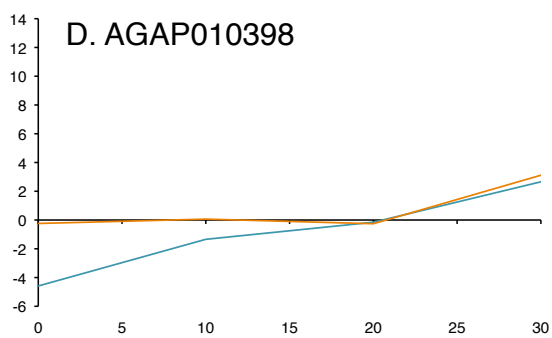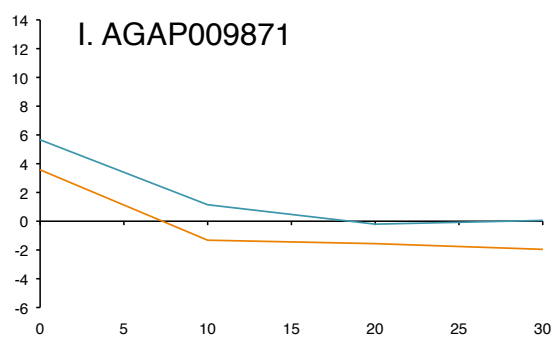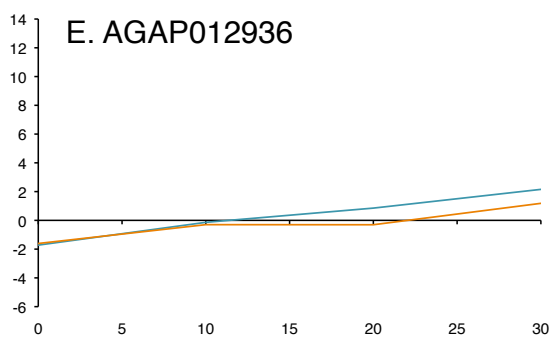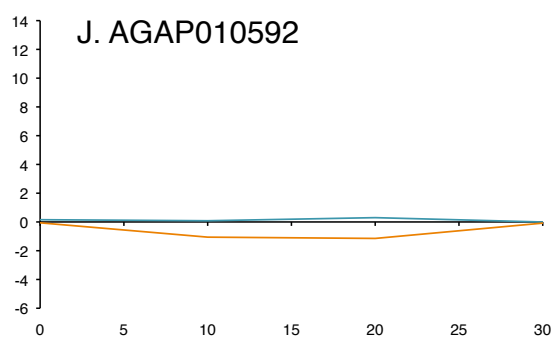

Age (days)

Supplement: Supplementary file 1 [file imb0019-0745-SD1.pdf]

Logcontrast of Ct values

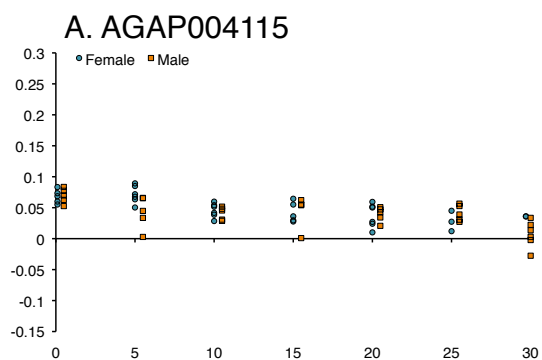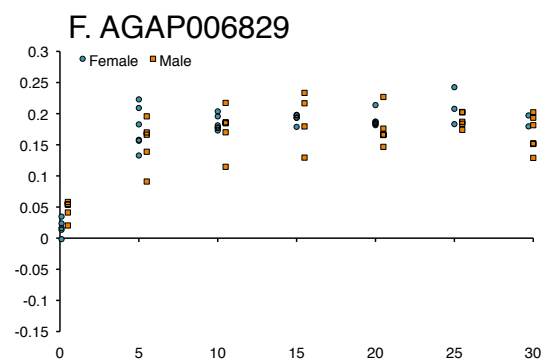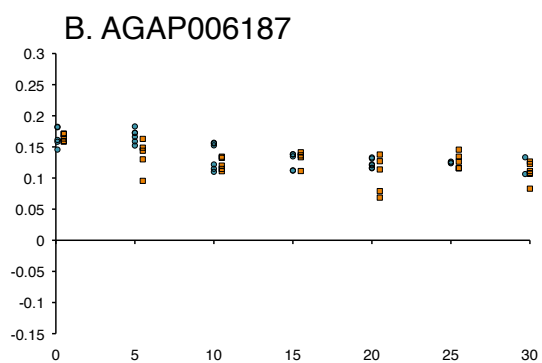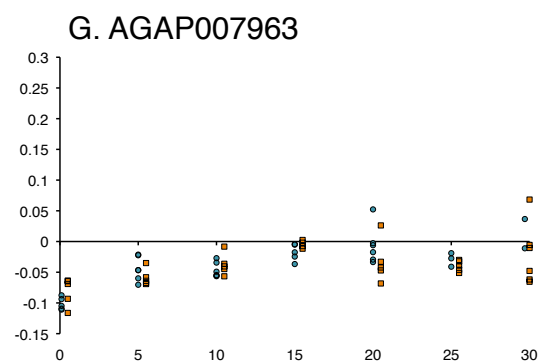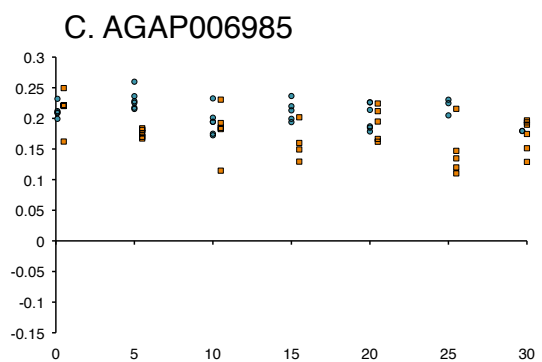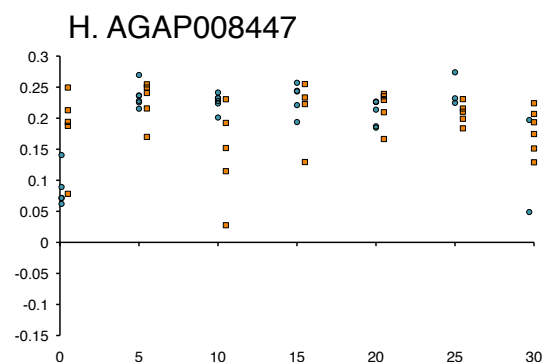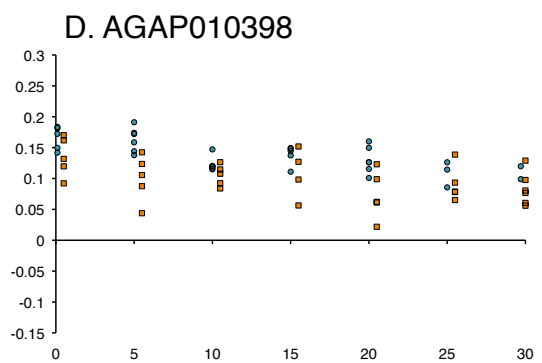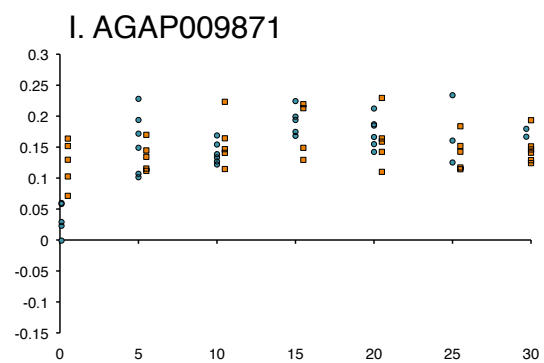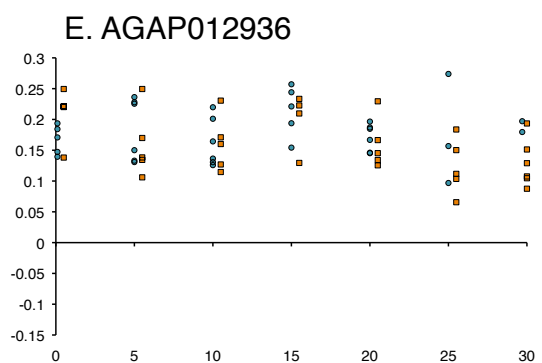

Age (days)

Supplement: Supplementary file 2 [file imb0019-0745-SD2.pdf]

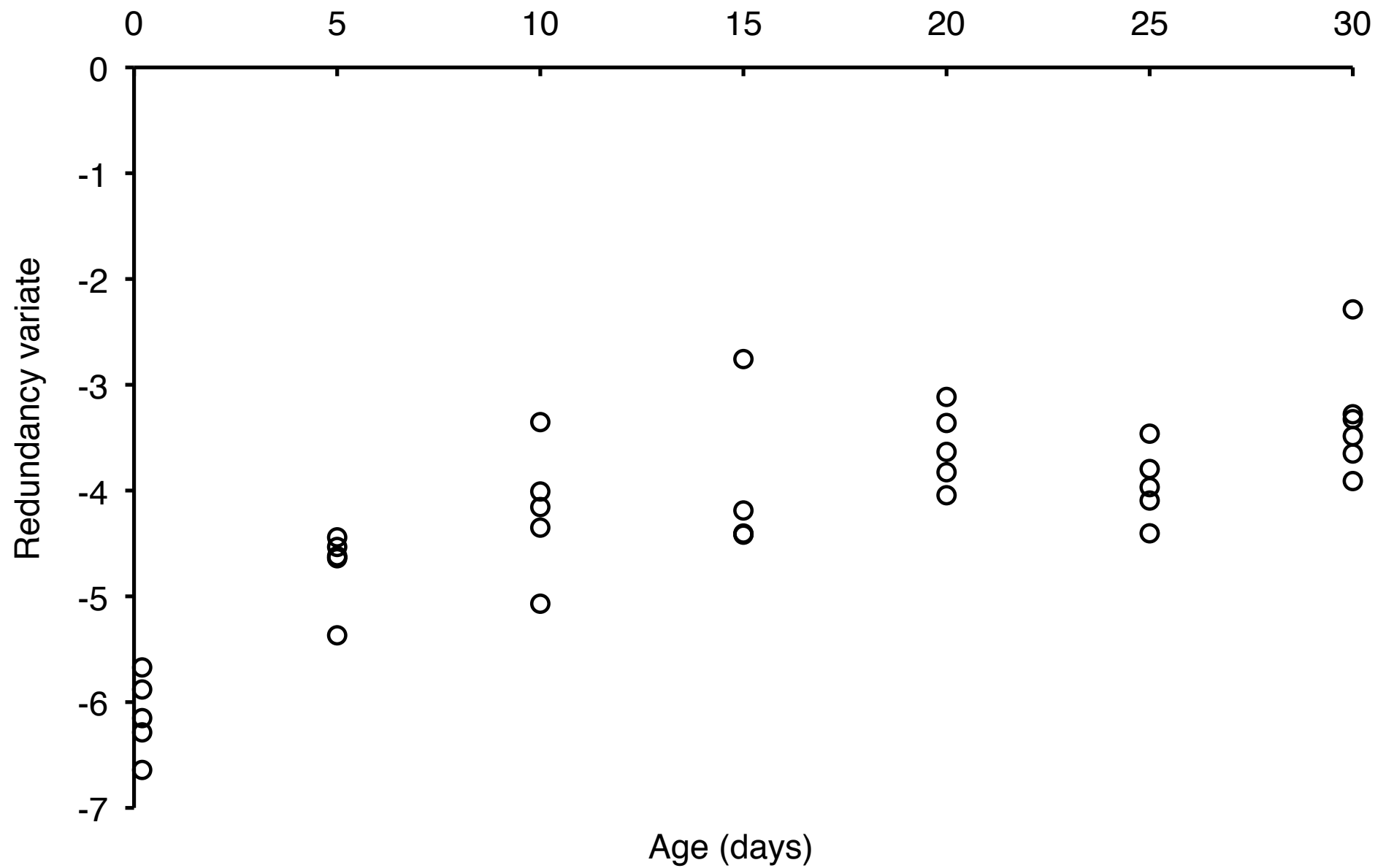

Supplement: Supplementary file 3 [file imb0019-0745-SD3.pdf]

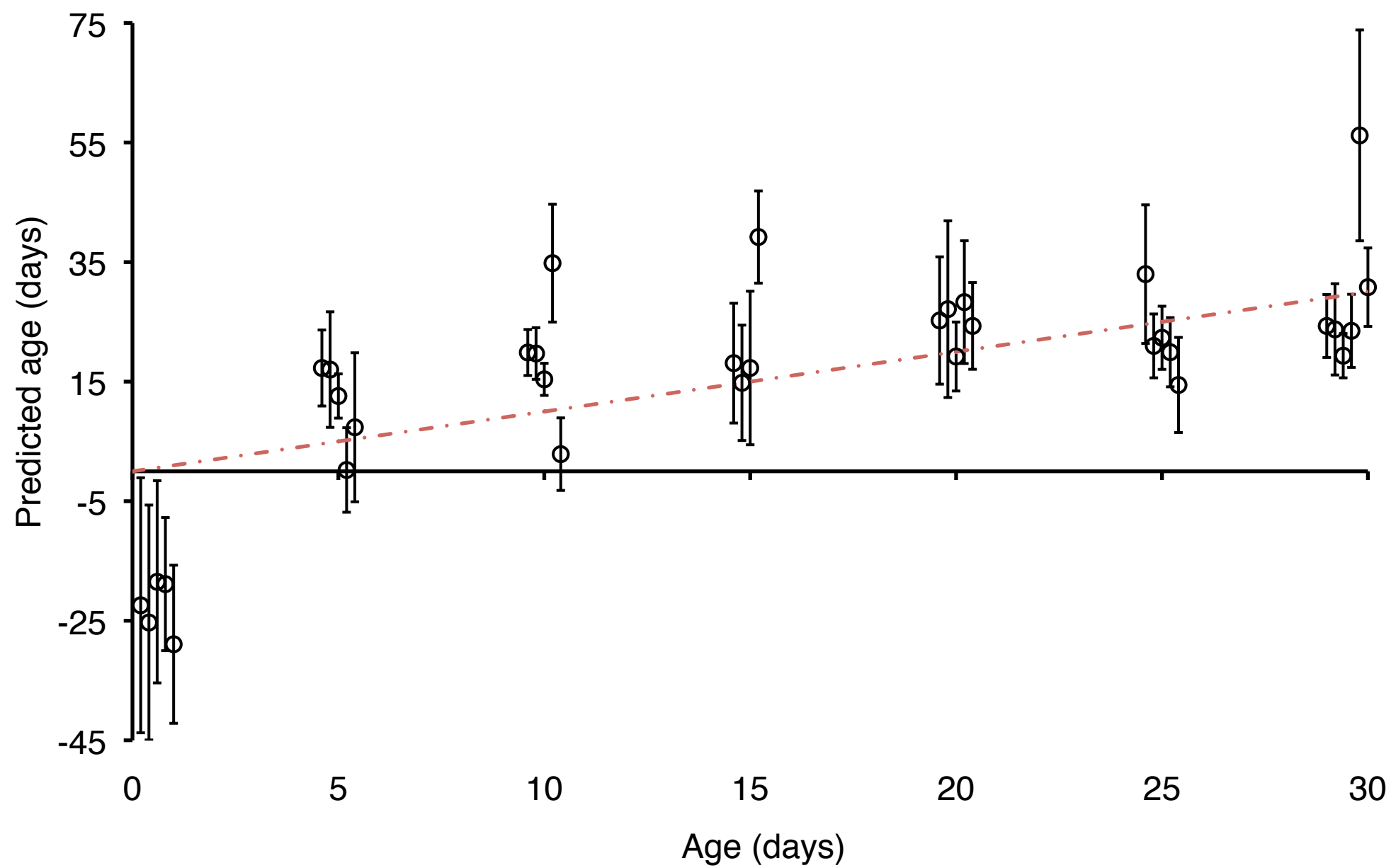

Supplement: Supplementary file 4 [file imb0019-0745-SD4.pdf]

A. Females

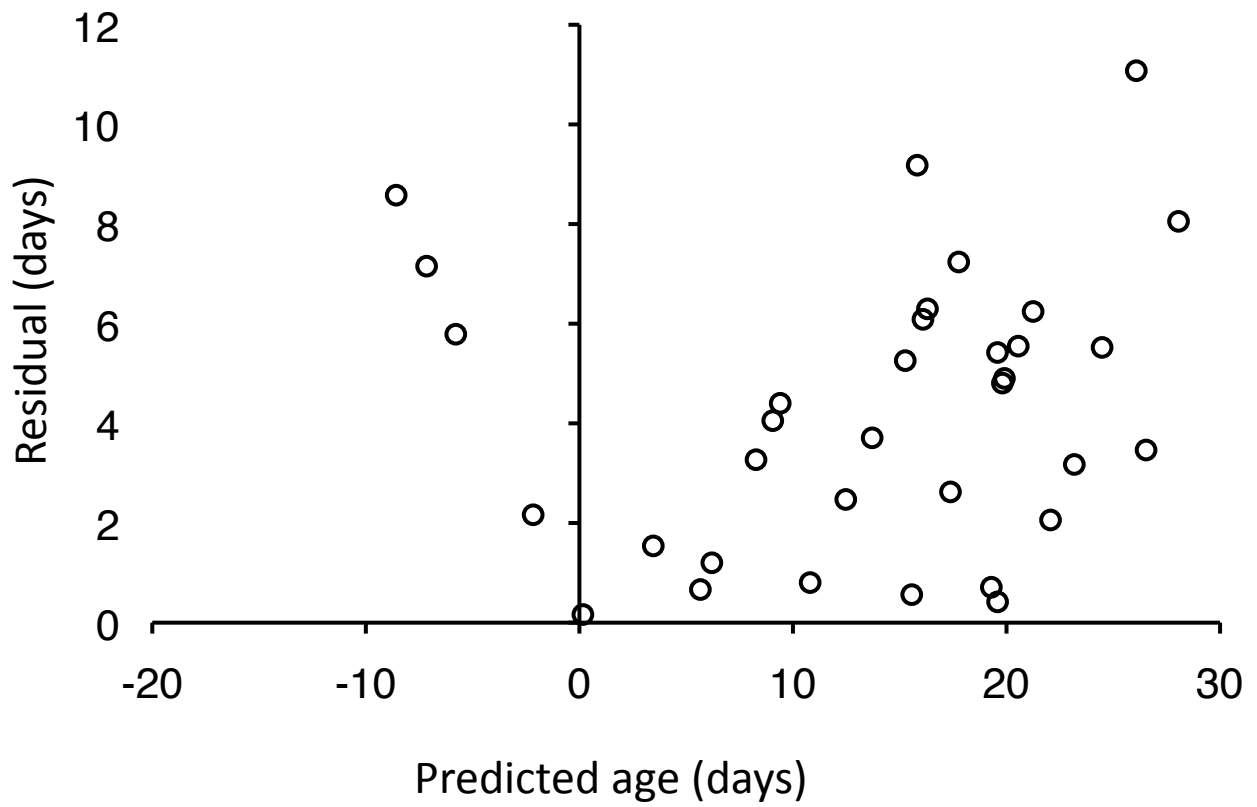

B. Males

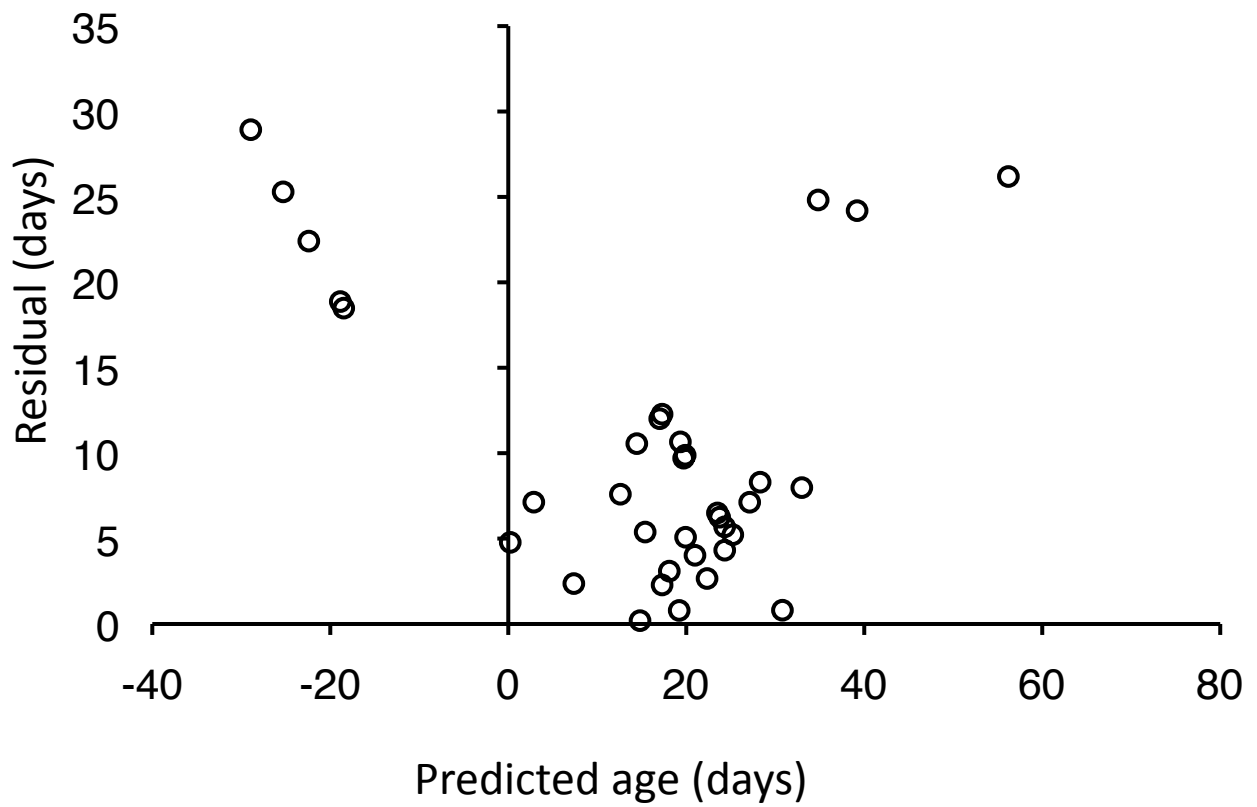

Supplement: Supplementary file 5 [file imb0019-0745-SD5.pdf]
